# Mr. Guthrie's Lectures on the Operative Surgery of the Eye

**Published:** 1823-09-01

**Authors:** 


*Lectures on the Operative Surgery of the Eye; being the Substance of that Part of the Course of Lectures on the Principles and Practice of Surgery which relates to the Diseases of that Organ. Published for the Purpose of assisting in bringing the Management of these Complaints within the Principles which regulate the Practice of Surgery in General.* By G. J. Guthrie, Deputy Inspector of Hospitals, &c. &c. &c. Octavo. pp. 500, five plates, coloured. London, August, 1823.

It is a well known fact that, previously to the year 1817, no lectures were delivered in Great Britain on the diseases of the eye, unless the concise and unsatisfactory observations made in the schools of anatomy on some of the operations be looked upon as such. The consequence was, that neither the surgeon nor the public considered a knowledge of this class of complaints as essentially necessary in the education of medical youths, and the treatment, of course, fell into the hands of the oculists, who made a good job of this oversight on the part of the general surgeon. But the various casualties of a protracted war in distant climates rendered it imperative on many of the physicians and surgeons of the army and navy to pay greater attention to the diseases of the eye, which impulse was greatly strengthened by the application and success of the late Mr. Saunders. From that time a gradual change has been operating in the minds of the profession, and the greater number are now convinced of the propriety of acquiring a thorough knowledge of the diseases of the eye by the same means, and in the same manner, as all other subjects in medicine and surgery are studied.

Great credit is due to Mr. Guthrie and his able colleague, Dr. Forbes, for instituting a course of lectures on the diseases in question, at the Eye-Infirmary in Mary-le-bone Street, where students in the West End of the Town may perfect themselves, not only in the theory but the practice of this important branch of surgery. The work before us comprises a part of these lectures, and is now published at the earnest desire of a great body of students, who were solicitous to have the means of reviewing the said prelections more at leisure, and when they would stand in need of their assistance in the course of their private practice afterwards. Mr. Guthrie, with great good sense, has avoided, as much as possible, entering into controversies—and where he has thought it right to differ in opinion with either the dead or the living, he has done so with that liberality and suavity which befit a learned and enlightened profession. Would to God that every member of it would make a firm determination to follow the same course!

We agree with Mr. Guthrie that it is an erroneous public opinion, kept up by those whom it concerns, that the organ of sight is of a very delicate nature, and so exquisitely sensible that it requires the greatest delicacy of touch—the utmost nicety of management. The regular students must be taught otherwise.

"They must learn, that the eye is not a delicate organ, that it will suffer more comparative violence, with less injury, than any other of importance in the whole body; that, so far from being exquisitely sensible, it is, when exposed in a healthy state, nearly the reverse, only becoming permanently so on the occurrence of inflammation; and that the ablest and most successful operators are neither the most gentle nor the most tender in their proceedings. The opinion of the exquisite sensibility of the eye has arisen from the pain which is felt on the admission of a small piece of dirt, or a fly, between the eye-lids; but this occurs from a wise and preservative provision of nature, on account of the insensibility of the eyeball itself. Let the eyelid be raised, and the same piece of dust applied to the surface of the eye, no pain, and scarcely a sensation, will be produced: remove the piece of dirt, turn out the lid, and whilst it is retained everted, place the piece of dirt upon it, no greater sensation will be induced than is felt when it is applied to the eyeball. The inference is, that both surfaces, when touched separately, are nearly insensible to this species of irritation. But let the same piece of dirt be put between the eyelid and the eyeball, and the sensation produced is exquisitely painful. To give rise to this sensation it is necessary that the two surfaces should come in contact, and that the foreign body be grasped between them. If this were not the case, an irreparable injury would often occur to the transparent part of the eye before it would be observed; and if the raising of the lid and the separation of the surfaces did not nearly annul sensation, an operation could not be performed for cataract; for, who could bear quietly the sensation which must arise from pushing a needle into the eye, if it were analogous to that arising from a fly or dry solid substance between the eye and the lids? The experiment may be tried in a very simple and conclusive manner by any one on himself, by merely keeping the lids apart by an effort of the will, when the end of the finger may be placed boldly on the eyeball without any inconvenience. Inflammation, by enlarging the vessels, gives rise to pain in the same way, and the sensation is at first as if some extraneous matter were interposed between the lids." *Preface*, vii.

The supposed difficulties attendant on the performance of operations on the eye partake of the same error, and have been kept up for the same reasons. Few persons, in fact, can estimate the liberties which may be taken with the eye until they have seen many operations on that organ, when the false ideas they may have imbibed will be completely removed, and new feelings will arise, in admiration of the wisdom and goodness of the divine architect.

The work commences with a long and very important lecture on INVERSION OF THE EYE-LIDS, containing a copious account of the opinions of ancient and modern authors, especially of the French and German schools. A perusal of this lecture will evince the great superiority of the English over both the abovementioned schools, as will be seen in the course of our analysis.

This disease has received various designations, as trichiasis, entropeon, phalangosis, ptosis, &c. according to the theories which have been formed of its nature and causes. "But the complaint is essentially the same under whatever name it may be characterized, differing perhaps only in degree." It consists in an inversion or irregular direction of one or more of the eyelashes—either alone or accompanied by an alteration of the natural curvature of the eyelid, in consequence of which the edge of the tarsus is directed inwards, and great irritation ensues from the unusual friction of the hairs on the eye-ball. Inflammation of the conjunctiva lining the palpebræ and covering the ball of the eye, is the first effect of an inversion of one or more hairs. This inflammation is, in time, communicated to the cornea, which becomes opake, and frequently ulcerates. When the disease has got into a chronic state, the integuments become of a redder colour than natural—the folds of skin usually observed at the upper part of the eyelid disappear—it is altogether thickened —and the cartilage assumes a vicious or unnatural curvature, most observable at the inner angle:—this, with the contraction of the conjunctiva at the commissures, and a slight ulceration or excoriation which frequently takes place at the outer canthus prevents the separation of the eyelids from being accomplished without considerable pain. Mr. Crampton, of Dublin, appears to have been the first to point out the principal cause of the disease (contraction of the angles of the lids) and also the proper *indication* of treatment. We say *indication*, for Mr. Guthrie does not quite coincide with that distinguished surgeon as to the precise *method* of cure.

"He (Mr. C.) first demonstrated that the levator palpebræ superioris was not inserted into the upper or convex margin of the tarsal cartilage, as was previously supposed, but into the parts connected with it and the conjunctiva; in consequence of which he lays considerable stress on the contraction and thickening of this part of the membrane being a principal cause of the inversion, and therefore recommends its division; but in this I cannot coincide, and suspect it has not the influence he attributes to it in the formation of this disease, and therefore conclude that the subsection, or division of it, which he recommends, is at least unnecessary, if not injurious. 10.

Mr. Guthrie is inclined to think, that "the inversion of the edge of the lid is more clearly dependent on the contraction of the angles, the undue action of the orbicularis muscle, and particularly that part of it called the musculus ciliaris, as well as on a vicious curvature of the cartilage." But these slight discrepancies of opinion we shall pass over, in order to quote the conclusions to which Mr. Guthrie's observations have led him.

"1. That the derangement of the eyelashes, constituting trichiasis, is frequently the consequence of disease; but seldom or never arises from unnatural formation, or from an accidental or too luxurient growth of the cilia.

2. The relaxation of the integuments, or a partial paralysis of the levator palpebræ, in a natural and otherwise healthy state of the eye and eyelids, are not primarily concerned in the formation of entropium, and never alone give rise to it; although, if other derangement take place, they may (by removing some power of resistance to it) assist in its more complete formation.

3. That, in a general or complete inversion of the edge of the tarsus of the upper lid, the swelling of the external parts and the spasmodic action of the orbicularis palpebrarum, first tend to the formation of the disease, and which is completely established by the contraction of the angles of the lids and the accidental inversion of such hairs as become stiff and matted by the vitiated discharge from the meibomian glands and conjunctiva.

"4. That the change in the curvature of the lid takes place principally from the contraction of the angles, whilst under the influence of the orbicularis, and not from the contraction of the conjunctiva, corresponding to the superior broad ligament, which supports the tarsus, and maintains the shape of the upper eyelid.

"These conclusions do not, of course, apply to such partial cases of trichiasis as result from the formation of a cicatrix, tumour, or other direct and obvious cause, to which I have already alluded. 15.

There are few diseases, in which so little alteration in the methods of operating has taken place since the days of Hippocrates, as in entropeon. The Father of Physic recommended a ligature to be passed through a fold of skin, by means of a needle, which, when tied, was allowed to remain until it ulcerated its way out. If this was not sufficient, the operation was to be repeated. Celsus advises two methods—the first, to burn the roots of the hairs with a red hot needle—the second, to raise and cut off a fold of skin, bringing the edges of the wound together by ligatures. A scarification was then to be made in the upper eyelid, under the roots of the lashes, that, being raised from the inferior part, they might point upwards. But, leaving the ancients, we shall descend to modern times: Scarpa contented himself with removing a portion of the skin of the eyelid. Beer recommends the evulsion of the hairs in the slightest cases, although he admits that he never effected a cure by it but in children; whilst, in the more severe and intractable instances, he advises the removal of a fold of skin, and the use of sutures. Weller says, if there be a deranged form of the edge of the eyelid, a radical cure will rarely be practicable, and confines his advice to the occasional removal of the offending hairs. Lastly, Jaeger recommends the removal of the edge of the eyelid, including the cilia, the detailed account of which operation Mr. Guthrie has extracted from a pamphlet of Dr. Hosp, and avers that it differs in nothing from the operation recommended by the late Mr. Saunders, and published many years before Jaeger first performed it.

"I have seen" says Mr. Guthrie, "three persons on whom this operation has been performed, and in two of them by Mr. Saunders. In all the deformity was considerable, and the relief obtained only partial.

"The operation of Dr. Jaeger being similar, the same objections of permanent deformity and imperfect cure apply to both; and a mutilation of this nature can indeed only be tolerated by comparison with the misery arising from the disease. Mr. Saunders admits this; and Dr. Jaeger, I presume, must do the same, and only adopts it because he is unacquainted with any operation that will always succeed without a mutilation of the part. What Mr. Saunders says, that no operation known previously to his time, not even that of Mr. Crampton, invariably succeeded, may be true; and therefore he conceived it to be an incurable disease, and to be relieved only by the excision of the part: but as the alterations I have made in Mr. Crampton's method render it in my opinion equal to the cure of every stage of the disease, I may be permitted to express my disapprobation of the operation recommended by Mr. Saunders and Dr. Jaeger; and this I am the more called upon to do, because there are some persons whose opinions deservedly carry great weight with them, who are generally correct in their observations, and whose sentiments on other points I respect, that still continue to recommend the operation of excision as performed by Mr. Saunders, as the only remedy in inveterate cases of entropium." 26.

If a hair turns inward without any discoverable cause, Mr. Guthrie coincides in the directions given by Mr. Travers, "that it must be kept plucked until, by the improved condition of the hair gland under the means used, the disease is removed." Mr. Travers also remarks that "if a case occurs in which the vitiated site or curvature of one or more cilia does not admit of correction, the corresponding follicle should be obliterated by repeatedly touching it with a fine caustic pencil." This method, Mr. Guthrie observes, is often effectual, and often ineffectual, inasmuch as it is apt to give rise to greater inconvenience from the cicatrixes which may be formed by its use.

"Where two or three hairs only turn in, with an irregularity of that part of the tarsus or contraction of the conjunctiva lining it, which may be perceived on turning it out, by a peculiar whiteness and thickening of the part, the operation described by Mr. Crampton ought to be preferred: 'Let the eyelid be well turned outwards by an assistant; let the operator then with a lancet divide the broad margin of the tarsus completely through by two perpendicular incisions, one on each side of the inverted hair or hairs; let him then, by a transverse section of the conjunctiva of the eyelid, unite the extremities of the perpendicular incisions. The portion of the cartilage contained within the incisions can then, if inverted with ease, be restored to its original situation." 29.

Mr. Crampton further suggests the propriety of completely insulating, in difficult cases, a small portion of the margin of the tarsus, leaving it connected to the eyelid by the external integument only. In cases of chronic inflammation, Mr. G. observes, where there is a commencing but incomplete general inversion of the cilia, the cure of the inflammation will be sufficient to restore the conjunctiva to its natural state, and the cilia to their original direction, without any especial means being employed for the cure of the commencing inversion; but when this proceeds too slowly, the application of the sulphuric acid, recommended by Helling and Quadri, should be resorted to, and will always be found sufficient. In many cases, Mr. G. thinks, in which there is little incurvation of the cartilage, and a moderate contraction of the angles, this plan will supersede the operation just now to be described.

"Quadri, who has written a quarto in praise of this method, and which he also liberally attributes to Helling of Berlin, applies it in the following manner: 1. A small quantity of concentrated sulphuric acid is to be applied, by means of a piece of smooth solid wood, to the centre of the affected part of the lid, and rubbed along on an oval space, a little exceeding in length the part on which the inverted hairs are situated, and from three to four or six lines in width, according to the inveteracy of the disease. The part ought to be wiped dry after the acid has been applied about ten seconds, in order to prevent any of it from getting into the eye.

"2. The application of the acid is now to be repeated, taking care that it may approach the edge of the lid, and touch the parts immediately over the inverted eyelashes; and it is to be continued or repeated a third or a fourth time, until the contraction of the parts draws the hairs from within outwards, or to their natural situation; when the operation is completed, and the part ought to be again thoroughly dried." 30.

We now come to the operation recommended by Mr. Guthrie himself.

"The operation I recommend, as equal to the cure of the worst cases, is to be performed in the following manner: The head being properly supported, the eyelids are to be gently separated; the patient is to be desired to refrain from making any effort whatsoever, and the surgeon is to wait until he sees that the lids are perfectly quiescent. A small narrow knife, or one blade of a blunt-pointed scissors, is then to be introduced close to the external angle, and a perpendicular incision made, of from a quarter, to half an inch in extent, or of a sufficient length to render the eyelid quite free; the quiescent state of the lids, and especially of the orbicularis muscle, enabling the surgeon to cut closer to the angle than he otherwise could do, and thus to divide the ligament, or at least the extremity of the cartilage. Another incision is to be made, in a similar way, at the inner angle; but this should not include the punctum lachrymale, as I have never found it necessary to do so; and, although the tears may continue to pass through the lateral canal into the sac, when the punctum has been included in the incision, they do not do so with equal freedom, and there is some observable deformity. The length to which the perpendicular incisions at both angles ought to extend, must now be decided upon by the appearance of the part; they must be continued, if necessary, by repeated touches with the scissors, until that part of the eyelid containing the tarsal cartilage is perfectly free, and is evidently not acted upon by the fibres of the orbicularis muscle, which lie upon it. This frequently causes the incisions, and especially the internal one, to be longer than is usually supposed to be necessary. The part included in the incisions is now to be completely everted, and retained by the fore finger of the operator's left hand against the brow of the patient; when, if any lateral attachment be observed, acting upon, and drawing, or confining the lid, it is to be divided, which is in fact still elongating the incision. On letting the eyelid fall on the eye, the edge of the tarsus and the hairs will frequently appear in the natural situation, in consequence of the relaxation of the angles, which bound them down; but if the tarsal cartilage has become altered in its curvature, this will be immediately perceived; it will turn inwards at its ciliary edge, and be completely bent at its extremities, more especially at the inner one, where it is more powerfully acted upon by the musculus ciliaris. On desiring the patient to raise the lid, he readily attempts it, but the action of the levator, in such cases of vicious curvature, causes the cartilage partly to resume its situation; and on examination, the curve will be observed to be so permanently vicious for about an eighth of an inch at each extremity, and especially at the inner, that it cannot be induced to resume its actual situation. When this is the case, the cartilage is to be divided, exactly at the place where it is bent, in its length, and in a direction at a right angle with the perpendicular incision. The portion thus slit is only connected with the common integuments of the eyelid; and although this incision scarcely exceeds one, and never two eighths of an inch, at both extremities, and in general is only necessary at the inner, it enables the surgeon to remove the altered curvature of the part. Mr. Crampton recommends that the two inner points of the perpendicular incisions should be now united by the subsection of Ætius and Paulus Ægineta, that is, by a division of the conjunctiva, which he supposes to be in a contracted state; but I have always found this division to be useless, and even injurious. It is useless, because it has no influence on the part, as a simple division of that kind unites or fills up in a very short time; and it is injurious, because, if made across the cartilage, it is apt to form a ridge or cicatrix which irritates the eye, and of which, among others, I have seen one particular instance. The operation being thus far accomplished, a fold of skin is to be cut away from that part of the eyelid included between the incisions; three or four ligatures are then to be introduced, and the divided parts, from which the fold has been removed, are to be neatly brought together by the ligatures, each of which ought to be twisted, and then fastened to the forehead by several short slips of sticking plaister, the ends being turned over the plaisters near the hair, and retained in that situation, to prevent their slipping. In raising the fold of skin, care should be taken to do it regularly with the fingers; it is also essential to the success of this part of the operation that it be done as close as possible to the margin of the eyelid. It may then be grasped by the forceps of Beer, which have transverse pieces, slightly curved for the purpose at their extremities, and close with a spring. The piece thus included, which need not be large, may be cut away at one or more strokes of a large pair of curved or straight scissors. The ligatures should be inserted first at each angle; and when the vicious curvature is considerable, I not only pass it through the skin, but take care that *the internal* one shall include, at its lower part, the *outer edge* of the margin of the eyelid, which, from its firmness, retains that ligature much longer than those which are passed through the skin only, and tends to prevent the possibility of a relapse. The ligatures thus placed are to be equally drawn up on the forehead, until the eyelid is *completely everted*, when they are to be fastened as directed. In order to prevent any attempt at union but by granulation, or a filling up of the incision, the edges are to be slightly touched with the sulphas cupri; the eye and eyelids are now to be carefully cleansed; a piece of lint, spread with the unguentum cetacei, is to be placed upon them; a small compress is to be put under the edge of the eye-bone and orbit; a retaining bandage covers the whole, and completes the different steps of the operation. When the disease is not considered of an inveterate nature, the ligatures may be introduced through the fold of skin, without cutting any portion away. They give little additional pain, and are infinitely more effectual than any other suspensorium with which I am acquainted, and they ought to be assisted by strips of adhesive plaister placed between them. The operation, accomplished with all the care I have described, will still fail, if equal attention be not daily paid to the subsequent dressing, on which, indeed, more depends than on the operation itself; so much, indeed, that I am disposed to consider inattention to it the most certain cause of failure. In more than seventy operations that I have performed, I have never known inflammation or other bad consequences ensue. The patient is to be kept perfectly quiet until the next morning, when the bandage and lint are to be removed, the eye carefully fomented and cleansed with warm water, and the dressings replaced. On the second day, great attention must be paid, that the ligatures keep the lid sufficiently raised; and if any union has taken place by adhesion at the angles of the incisions, it must be broken through with the probe. On the third day the plaisters attaching the ligatures to the forehead, will in general require to be exchanged. The ligatures themselves must be supported by straps of plaister placed vertically between them; the edges of the incisions should again be touched with the sulphas cupri, or separated by the probe. The great art of the cure now consists in causing the incisions to be filled up by granulations only, so that the eyelid may be lengthened as much as possible, and which can only be effected by a continuance of the means indicated. In a few days more, and especially by the continued elevation of the lid, the ligatures cut their way out, during which period the eyelid is gradually lowered, and by the time the incisions have filled up, it will have resumed its natural situation, and the cure will be completed. There will, in general, be an indentation in the ciliary margin of the tarsus, where the incisions were made, but this is sometimes scarcely perceptible and never detrimental. If the patient should have kept the lids closed at the commencement of the operation by the action of the orbicularis, the first incision may not have been made as close as might be desired to the angle of the lids; and in this case a hair or two at that part may not afterwards obtain quite its, or their original direction, and cause some inconvenience; but this does not often happen, as these hairs are usually carried clear of the eye.

"The operation on the under eyelid is analogous to that on the upper, but is less severe, as the parts are more simple. It consists, in inveterate cases, of a perpendicular incision made with the scissors at the outer angle, and carried downwards to such extent as will perfectly relieve the inversion. This incision, by dividing the fibres of the orbicularis at that part as well as the conjunctiva, renders the muscular fibres powerless; and when it is properly made, the patient is incapable of moving the lid. One ligature is now to be inserted at the margin of the lid, and the threads fastened below the jaw by sticking plaister, so as to keep the eyelid everted." 35.

The subsequent treatment of the eye is much less than might be supposed. The principal cause of irritation being removed, the inflammation gradually subsides, the conjunctiva daily assumes a more natural appearance, the opacities of the cornea diminish. Mild stimulants, such as Battley's liquor opii sedativus, the liq. plumb. sub. the solut. argent. nit. (two to four grains to the ounce) in distilled water, are the best applications after the incisions are healed.

"Where the conjunctiva is thickened, or granulated, or fungous, the latter remedy dropped into the eye twice a day is very useful; and in more inveterate cases, a saturated solution of the sulphas cupri in distilled water applied with a camel's hair brush, and well washed off with plain water, is often of essential service. In every case, some mild ointment should be applied to the edges of the lids at night, and any secretion which may have arisen and become hard, should be removed by the aid of warm water in the morning. Indeed, the lids, whilst any vitiated secretion remains, should be carefully examined *every* morning, and great attention paid that any hair which has changed its direction from having been clogged by the discharge, be replaced in its situation, or pulled out if it appears to resume its place with difficulty. The best ointments are, the ung. cetacei, the ung. zinci oxydi, the ointment of Janin, the ung. hydr. albi gr. 2 ad ℥j. the ung. hydr. nitr. et oxydi rubri, six times reduced with the ung. cetacei, assisted during the day by the occasional use of any of the mild astringent lotions." 37.

By the above means, Mr. Guthrie avers that a disease which has been an opprobrium to surgery since the days of Hippocrates, may be always cured, under whatever name or description it may be included. Six illustrative cases are appended which we recommend to the serious and attentive perusal of the ophthalmic practitioner. Upon the whole, this section of Mr. Guthrie's work is exceedingly interesting —contains much originality—and evinces great power of discrimination, with no want of critical acumen.

### RELAXATION OF THE UPPER LID.

This is, for the most part, a symptomatic disease, and frequently follows as a consequence of apoplexy, being accompanied by hemiplegia or more partial paralysis. From this it is evident, that few cases can be bettered by any surgical operation—in fact, only those which are really the consequence of a relaxation of the skin and integuments of the lid from previous distention, and combined with atony of the levator palpebræ—or when the disease is congenital. The operation which has been found useful is, that for trichiasis, the removal of a fold of skin from the eyelid, whereby a diminution of the length of the integument takes place, counterbalancing the relaxation which had previously occurred. If, however, the levator palpebræ be in a state of atony, the operation can only be partially successful.

"In trichiasis, the skin removed should always be immediately from over the tarsal cartilage, whilst in a case of pure relaxation it should be in that folding part of the integuments above the upper edge of the cartilage, or nearer to the eyebrow. Scarpa considers sutures as unnecessary, and advises the edges of the wound to be brought into contact and retained by means of strips of adhesive plaister; but especially by applying a compress upon the supercilium, and another upon the inferior edge of the orbit, and over these a uniting bandage. I am disposed, however, to insist on the use of sutures, and I have reason to believe they assist materially in effecting a more perfect cure. If the atony of the levator palpebræ should depend on mere debility from inaction, the suspension of the lid will be sufficient with the attempts at motion of the patient, to effect a cure, which will also be assisted by slight stimulant applications; but if, on the contrary, there be a real paralysis of this muscle, I do not see how the operation can do more than raise the eyelid to a certain extent, so as to remove a part of the deformity, and the cure of the paralysis must be attempted according to the nature of the immediate cause. Indeed, in such a case the operation should follow rather than precede the general treatment." 43.

Cases in elucidation are detailed by our author.

### EVERSION OF THE EYELIDS.—ECTROPEON.

This disease consists in a turning outwards of the upper or under eyelid. It is much less frequent than the opposite disease, (entropeon,) and but rarely affects the upper eyelid. The symptoms and appearances of the part depend entirely on the nature and causes of the disease, and differ from each other in a remarkable degree, demanding imperatively a regulated method of cure. Inattention to this has given rise to operations, and the administration of remedies that were very inapplicable. In order, then, to elucidate this subject, our author has deemed it adviseable to regard ectropium as of four different species, or, at least, as produced in four different ways.

"1. As depending on chronic inflammation, accompanied by contraction of the skin and of the integuments of the lid; but without any marked cicatrix.

"2. As depending on acute inflammation, or immediately following it, with relaxation or swelling of the conjunctiva.

"3. As depending on the contraction occasioned by a cicatrix, on the healing of a wound, on, or in, the immediate vicinity of the eyelid.

"4. On paralysis.

"This disease, in whatsoever way it may be induced, is never productive of so much distress to the patient as the inversion of the lid. It is in general a complaint of inconvenience, and of annoyance from its unsightliness, rather than of constant pain; and as the eye is seldom much implicated by it, unless it be of long standing, or of great severity, the sufferer, if a poor man, is capable of following his occupation for months and even years, before the extension of the disease to the eyeball causes a sufficient obstacle to prevent it. At first there is merely a dropping (stillicidium) of the tears, and an increased and vitiated discharge from the irritated glands of the eyelids; this is accompanied by excoriation and redness of the skin of the cheek, and is followed by a slight defect in vision, resulting from the thickened secretions which pass over the cornea, as well as from the greater exposure of the conjunctiva covering it. The conjunctiva of the eyeball is not always inflamed, neither is that lining the eyelid necessarily thickened, unless in certain states, to be hereafter described: whilst in others, the eyeball may not only be inflamed, but, in a subsequent stage, the cornea may be rendered opaque, and the conjunctiva covering it thickened, dried, and withered, so as totally to alter the natural appearance of the eye. The conjunctiva, forming the fold between the eyeball and the lower lid, may be so swelled and thickened, as even to form an almost horny substance between them; whilst, in other cases, the whole of the conjunctiva palpebræ may protrude, so as to hide the ball of the eye, and present a most hideous object to the spectator; a good representation of which has been given by Dr. Vetch in the fourth figure of his second plate." 51.

This disease was well known to the ancients. Hippocrates alludes to one cause of it, viz. a fungous state of the eyelids, and directs the thickened part to be removed with the knife, the cut surface to be cauterized. Celsus, considering it as a consequence of the removal of too much skin in the operation for entropeon, says, "when too much of the eyelid is wanting, (alluding to the upper,) there is no remedy for it; but if a small part only, then it may be cured by making an arched incision, with the ends upwards, a little below the eyebrow, but not so deep as to injure the cartilage." The cure is then to be completed by granulation. Etius (from Antyllus) has improved on the operation of Celsus; but we cannot pursue the historical part of the work before us. We hasten, therefore, to lay before our readers the practical directions of Mr. Guthrie himself.

"The first kind of disease, or that which is dependent on chronic inflammation, usually takes place after the long continuance of that form of it, which has obtained the name of lippitudo, and prevails more or less in almost every case of it of long standing, both in the child and the adult. In this complaint, which begins as an inflammation of the meibomian glands, extending to the conjunctiva palpebræ and the edges of the eyelids, we find, after a time, that the disease assumes a chronic form; the itching and pain diminish; the redness of the conjunctiva subsides; it becomes smoother, of a yellower colour, and the vessels are more distinctly marked; small ulcerations have formed at the roots of the hairs; the edge of the lid becomes thicker and of a more shining reddish colour than natural, and is also excoriated in various parts; the eyelashes have mostly fallen out; the secretion from the meibomian glands is considerably augmented in quantity and altered in quality, it exudes and passes over the edge of the eyelid with the tears, which the irritation of the eye, from any exciting cause, produces in greater quantity than can be carried off by the puncta lachrymalia. In poor persons, this matter dries and forms crusts at the roots of the hairs, glues the eyes together during the night, and is a constant source of irritation to the skin of the eyelid and cheek. These parts become in turn excoriated, are frequently studded with small spots of ulceration, and the skin loses in consequence its natural pliability, becoming hardened, irritable, and contracted. If the eye be examined when in this state, the remaining cilia of the lower lid will be found to turn downwards. and the edge of the eyelid within the hairs, and where the ducts of the meibomian glands open, will be so much turned outwards as to show the reddish yellow conjunctiva lining the palpebra, and giving the peculiar appearance to the eye, which characterizes this complaint. If the eye be irritated by a high wind or exposure to cold, the tears stand upon the edge of the lid, or fall over, constituting what is often called a blear eye. This partial, or rather commencing eversion of the eyelid is only so far a necessary consequence of the disease as it depends on the excoriation, contraction, and hardening of the skin, the result of the passage of the vitiated secretions over it, and which, by drying on it, increase the irritation. That this is the cause of the eversion, is demonstrated by the simple experiment of pressing on the skin so as to draw it downwards, or increase the contraction, when the eyelid becomes immediately everted. That this artificial assistance is merely adding what occurs when the disease is in a more advanced stage, is proved by the fact of the eversion being more complete when the contraction is greater; and after it has proceeded so far as to turn the cartilage outwards, the firmness and elasticity of which opposes at first a powerful resistance, the complete eversion is promptly effected; for the external contraction is now only opposed by the ligaments uniting the extremities of the tarsal cartilages to the angles of the eyelid, which have been gradually yielding, and are no longer capable of offering any effectual counterpoise to the increasing power acquired by the contracting skin. That this view of the subject is correct, I am in the habit of proving almost every week at the Infirmary, by contrasting the different stages to the gentlemen attending; when that which may not be so apparent on the inspection of a single case, is clearly demonstrated on the examination of several. The altered appearance of the skin is also strongly confirmative of the opinion. In the first state of the first species, it is only rough, hardened, and slightly contracted. In the second, it is more contracted and apparently deficient in quantity; the lid can, however, be restored to its natural position by pressure made with the finger at the external angle. In the last stage, however, or that of complete eversion, the deficiency of skin, from its contraction, is so evident as not to be overlooked, and the eyelid cannot be restored to its situation by the finger in many instances, whilst it is only accomplished with difficulty in all.

"The edge of the lower lid is not straight when in its natural situation, but has a gentle curvature corresponding to the eyeball, which becomes more apparent, and is perhaps slightly increased, when completely everted, from being drawn downwards in the centre; and from the inferior ligament and the conjunctiva offering less resistance at that part than at any other: whilst at the angles the alteration is infinitely greater, so much so, that at the outer angle, the eversion does not gradually commence, but is suddenly effected, as if the ligament at that part was twisted rather than stretched. It is usual to consider this disease as dependent on an elongation of the tarsus; which, if it mean the tarsal cartilage, or that part of the lid which receives its shape or form from it, and on the outer edge of which the hairs are situated, is incorrect; the structure of the cartilage not admitting of such extension, and the only part which can be elongated is that portion of the eyelid, at the two extremities of the cartilage, which unite it to the upper lid, and form the inner and outer angles. This union, which is a ligamentous one, is so very short at the outer angle, as to admit of little stretching: and we find accordingly, that when complete eversion takes place, it is effected by a twisting at this part, rather than by an elongation. The cartilage, however, in consequence of the unnatural direction it has been forced to take, assumes a different or vicious curvature, which is more or less observable according to the length of time the disease has existed. It is this alteration of curvature which causes the lid to stand out from the eye, and appear to be elongated, when the eversion is suddenly overcome by operation or otherwise, and has therefore led to what I conceive to be, in the two first species of this disease, an error, viz. cutting out a portion of the lid, and forcibly altering, by ligature, the curvature of the part, instead of leaving this process to the efforts of nature, assisted in a gentle manner by art. That these are sufficient in the more simple states of disease, is proved, by the fact that a too long continuance of the means employed will ultimately give rise to a disease of an exactly opposite nature, a case of which I shall hereafter relate." 57.

Here Mr. Guthrie enters on a critical examination of some positions of Scarpa relating to the subject of ectropeon, which we cannot follow very closely. Scarpa appears to regard the disease as of two distinct species, as far as regards the causes—"the one arising from a preternatural tumefaction of the palpebra, which not only separates its edge from the eyeball, but also presses upon it in such a manner, as ultimately to evert it—the other produced by a shortening of the skin covering the eyelid, or that of the neighbouring parts, by which the ciliary edge is, in the first instance, separated from the ball of the eye, and afterwards gradually turned outwards, together with the whole of the eyelid." This latter species of eversion appears to be that described by Mr.

Guthrie; but Scarpa seems to consider this species as generally incurable, inasmuch as the principal cause consists in the loss of a portion of the skin of the eyelid or surrounding parts, which no artifice hitherto known can restore.

" This quotation," says Mr. Guthrie, " proves that Scarpa was not aware of, or did not think it necessary to notice, that species which I have just described; but my object in referring to his work is not directed to this point, but to that part of his statement in which he says, 'this effect (the eversion) no sooner takes place, than it is succeeded by another no less inconvenient, the tumefaction of the internal membrane of the eyelid, which also greatly contributes to complete the eversion,' &c. &c. That this is not a constant occurrence, I have no hesitation in affirming, and that it is, on the contrary, a rare one in the species I have referred to; a fact which, while it shows the propriety of the division I have made, is also of importance, as it influences materially the mode of cure; and the first four cases to be related are incontrovertible proofs establishing the correctness of my opinion. In denying, then, the fungous state of the conjunctiva, I by no means intend to imply that it retains its natural characters without alteration. I have, on the contrary, noticed its primary changes; and I have now to state, that as the eversion takes place, the exposed and everted conjunctiva becomes thickened, of a more general yellowish red colour, but not granulated, and is constantly bedewed with the tears and vitiated secretions of the adjacent parts. The thickening is not such, however, as to enable an operator to remove it with a knife or scissors, or to render such proceeding necessary, for it subsides with the removal of the complaint. According to the views I have given of this species of disease, the indications of cure are two; 1st, to relieve the contraction of the skin externally; 2d, to restore and retain the lid in its proper situation, until the unnatural curvature of the cartilage has been overcome, and the chronic inflammation removed; when the cure will be completed. The first indication is in some measure fulfilled by washing the external parts with warm water, so as to leave the skin as clean as possible. It is then to be carefully dried and repeatedly anointed with the unguentum zinci oxydi, for three or four days, so as to form a covering which shall protect it from the matters which usually pass over it. It is necessary here to bring to our recollection, that the skin is merely contracted from irritation; and is disposed, from the inherent properties of our nature, to resume its natural appearance on the subsidence of the cause of irritation. It is in a different state from that forming the cicatrix of an ulcer; which, although it be called skin, is in reality only a smooth membrane, in some respects resembling it, but possessing neither its furrows, nor, of course, its great extensibility, whilst it is equally destitute of the elasticity depending on continuity of structure. The skin, thus situated and relieved from irritation, becomes softer and more pliable, ceases to contract, and, if it does not even relax, is at least in a favourable state to yield to a mild extension. This is accomplished, and with it the second indication, by the application of the sulphuric acid of the shops, in the following manner. The lid having been previously cleansed, so as to prevent its slipping, the conjunctiva is to be gently wiped dry, and everted as much as possible, so as to show the part where it begins to be reflected over the eyeball. The upper eyelid ought to be a little raised by the finger of an assistant, and the patient should be desired to look upwards. The blunt end of a common silver probe is then to be dipped into some sulphuric acid, and rubbed with its side flat upon the conjunctiva, so that every part may be touched by the acid. The round point of the probe should be carried as far as where the reflection to the ball begins, but that part of the conjunctiva which covers it should be preserved inviolate. The punctum lachrymale, caruncle, and semilunar fold, are also to be avoided; but the external angle, as well as every other part, must be carefully rubbed. The effect of the acid will be observed by the conjunctiva turning white where it has been touched by it; and in order to prevent the acid from affecting the eyeball, a stream of water should now be directed over the eyelid by an elastic gum syringe. If the acid should not appear to have turned the conjunctiva sufficiently white, it may be repeated with the same precautions; and if the patient washes the eye afterwards in cold water, no inconvenience will result, the pain is comparatively trifling, and very few persons complain of it. The application of the acid should be repeated every fourth day; and when applied in the manner directed, it does not cause a slough, but a general contraction of the part, which is, however, only perceptible after two or three applications, by its effect in inverting the lid, which gradually begins to take place. After six or eight applications the cure will be more than half accomplished, and in most cases of this species of eversion, the thickening of the conjunctiva will have subsided; if the operator should have become bolder in the use of the acid, slight ulcerations may be produced, but these are favourable to the completion of the cure. The ung. zinci oxydi is to be constantly applied to the skin, and the ung. hydr. nitrati, diminished in strength by the admixture of the ung. cetacei, in the proportion of four or six parts to one, to the edge of the lids and roots of the hairs every night, so as to produce a moderate sensation of smarting.

"The immediate effect of the acid is not confined to, neither does it cease in four days, but remains for a much longer period; so that if the use of it be continued until the eyelid be entirely restored to its proper situation, the continuance of its action in producing contraction will cause an inversion of the eyelid, of which I give an instance in Case III. and whence the precaution now to be observed, of a longer interval between the applications of the acid, after the lid has two-thirds recovered from the state of eversion, in order to prevent such an accident taking place. The occurrence of it is, however, a valuable fact, as it proves the efficacy of the practice, and the power of a remedy, which if it be capable, when too frequently or too violently applied, of causing an inversion of the eyelid, must, if correctly employed, be sufficient to restore it to its natural state." 62.

Of the second species, or that dependent on acute inflammation, or immediately following it with relaxation or swelling of the conjunctiva, Dr. Vetch has given a very excellent description, at page 228 *et seq.* of his work, which is quoted by Mr. Guthrie.

In young children, our author observes, an eversion of one or both eyelids is not an infrequent occurrence during the purulent inflammation of the parts—especially on any attempt being made to examine the eyes whilst the child is crying. This eversion can always be relieved at the moment, by taking hold of the eyelid at each angle with the finger and thumb, and turning it inwards, whilst, or after it has been drawn downwards. Passing over some observations on the practice of foreign ophthalmologists, we come to that of Sir William Adams. This gentleman having found the methods then in use for the cure of ectropium insufficient, was induced to try a new method, by cutting out a portion of the eyelid in the shape of the letter V, of which practice he details four successful cases—all of the second species. Mr. Guthrie, after quoting Sir William's directions for the operation, makes the following remarks:—

"This operation is founded on the idea of the eyelid being elongated beyond the power of recovery, either by the assisted or unassisted efforts of nature, and therefore requiring to be shortened to restore it to its proper length. This supposition, in regard to the two first species, is certainly erroneous; for four-fifths of the eyelid cannot be elongated, and the remaining fifth part is only so partially stretched, in these cases, as not to form an irremediable obstacle to the retention of the part in its natural position. That it does not do so, I have given a positive proof, in showing that the opposite state of disease may be readily induced; and Beer, whose authority must be decisive, at page 137 of his first volume, when on this subject, gives an express caution not to apply the lapis infernalis too frequently, or an inversion of the lid will be the consequence. According, then, to the principles which regulate the practice of surgery in all other cases of disease, this operation ought not to be performed in either of the two first species, as the same effects may be produced by more simple means; with little pain, without one day's confinement, and certainly with less deformity, (even if it be merely temporary,) inasmuch as there will not be an external incision. In this opinion I am not singular; for Mr. Samuel Cooper has quoted, in his Dictionary of Surgery, the following observation of Mons. Roux on this subject: 'What Sir W. Adams says, with a view of enhancing the value of his own method, about the frequent recurrence of ectropium, when the conjunctiva is simply cut out, is a gratuitous assertion, contradicted by experience. I have already in a great number of cases, undertaken the cure of ectropium in the common way: the operation always succeeded as much as the degree or other circumstances of the disease allowed; and I have not yet observed an instance of a relapse.'

"It will be admitted by every one, that, as the second species of eversion essentially consists in a fungous or granulated state of the conjunctiva, with, for the most part, a swelling, either sensible or insensible, at the angle, where the membrane passes from the eyelid to the ball of the eye, and which is even said to be, with a contracted state of the external skin, in some cases the cause of the eversion; the indications of cure must be, first, to remove the diseased growth or state of the conjunctiva; secondly, to restore the lid to its natural situation.

"In order to accomplish the first indication, recourse must be had to the knife, the scissors, or such remedies as, acting partly as caustics, partly as stimulants, gradually restore the conjunctiva to its natural state. When this membrane is either fungous or granulated, I may, I believe, say, without entering into particulars, that it is now very generally admitted that the knife and scissors are the most objectionable means of cure, and are nearly, if not altogether, abandoned; which at once establishes the propriety of the practice of the ancients, by stimulants and caustics; and, among our contemporaries, of Beer and Scarpa in particular. I am, however, disposed to prefer the use of sulphuric acid, every four days, and a gentle application of the sulphate of copper, daily, or every second day, to the means recommended by either of them; and I have no hesitation in affirming, that the cure effected by these means, in addition to those minor remedies I have already noticed for the cure of the first species, will be complete and permanent. This point being settled, the only remaining question for consideration is, whether the cure can be accomplished in less time by the operation of cutting out a portion of the eyelid, than by the treatment more usually recommended. To judge only from the statements and cases which have been published, the answer would be decidedly in the affirmative, as they would lead to the belief that every thing was done by the scissors, both in relation to the cutting out of the angular portion, and the removal of the fungous or granulated state of the conjunctiva. But we have the authority of Sir W. Adams for believing, that the method of removing a granulated surface of the eyelid by the scissors, as recommended by Mr. Saunders, is incompetent to the purpose, more especially in the lower lid; and he introduced a knife in order to supersede the scissors, which knife has properly yielded to the use of escharotics. On these points, and on the incompetence of the scissors, I perfectly agree; and therefore draw what I conceive to be a correct inference, that if the scissors are not competent for the purpose intended at one time, they are equally unfit, in the same kind of case and under similar circumstances, at another. When the fold of conjunctiva is the principal cause of eversion, and has become hard or horny, it ought to be removed by the scissors, and a compress and bandage should be applied over the eye; but as the scissors never cut away the whole of a granulated surface, it must be subsequently restored to its natural state by the operations of nature, either alone or assisted by stimulants or caustics, in the same manner as in the preceding states of disease; which fact being understood, places the two methods on a par. If there be, however, no fungous conjunctiva lining the cartilage, and merely a hardened swelling or fold at its angle of union with the eyeball, then the operation will be more advantageous in regard to time.

"If, in judging this operation by the general principle of surgery, I have been obliged to reject it in the two first species of this complaint, I by no means wish to condemn it in all; and I have now to show, in the third species, in what way it may be highly useful." 71.

This third species, or that caused by a contraction from cicatrix, has always been considered as a very intractable complaint, and only curable when the eversion is not complete, and the cicatrix not extensive nor firmly attached to the bone beneath.

"In this species, the appearances and actual situation of parts differ materially from the other two, and are influenced by different circumstances. It is well known, that the first step towards the cure of an ulcer or wound, is the drawing in of the surrounding parts, in a manner commensurate with the growth of the granulations, which are to fill up the vacuity that has been made by the progress of the ulcer; and by the time cicatrization has taken place, the cicatrix bears no proportion to the size of the original wound. But this process of contraction does not stop here; it continues after the ulcer has healed, especially in loose parts; and, in burns of the neck, it is capable of drawing down and fastening the chin to the breast. This diminution in the quantity of new-formed parts continues until there is little left to mark where the solution of continuity existed: but this little does not resemble the original structure; it is firmer, harder, more stringy, and somewhat resembling the nature of ligament. The cure has been effected at the expense of the surrounding parts, which if loose, as in the neck or in the eyelid, may be drawn into a very considerable extent. In consequence of a knowledge of this fact, our attention, in cases of burns in the neck, should be invariably directed to the position of the head, which must be kept backwards until long after the wound has healed, in order to counteract, as much and as mechanically as possible, this tendency to contraction, and to force Nature to form more new skin, or a larger cicatrix, which she is always very unwilling to do, and is often indeed incapable of doing. The same thing takes place on the eyelid; and if an ulcer be allowed to heal quickly, eversion, in a greater or less degree, is an invariable consequence. In the first and second species, the operation of this cause is slight and on the edge of the eyelid, or cartilage; in the third, it is greater and on the whole external surface of the cartilage, which is turned downwards, whilst the eyeball is more exposed; the whole of the conjunctiva of the under lid is distinctly seen, and forms something resembling an inclined plane from its angle of reflection over the eyeball, outwards and downwards. The deformity is more striking. In the second and third species, the conjunctiva always forms more or less of a sulcus, or hollow, with the eyeball at the angle of reflection: in the third, this part is fully exposed, the conjunctiva is drawn downwards; the tears always flow over the cheek; the curvature of the tarsus is altered; the ligaments are more stretched, and the elongation of the eyelid becomes more apparent; the conjunctiva is sometimes fungous, or granulated, and occasionally but little affected, or altered, but in many instances it assumes a lippitudinous appearance; the cicatrix is hard, and for the most part, firmly attached to the cheek-bone." 73.

It was for the cure of this species that Bordenave recommended the double operation of the removal of the cicatrix and of the fold of the conjunctiva, which, although not successful in effecting a perfect cure, was productive of much relief to his patients. It was, after all, only returning in some measure to the practice of the ancients. That it was not sufficient, is attested by Scarpa and Beer—and that the German ophthalmologists have not hitherto done much towards relieving it is shewn by a case related by Professor Dzondi, of Hallé, and now published by Mr. Guthrie, at page 73 of the present work.

"To effect a cure of this species of eversion, the indications are two in number: first, to remove the cicatrix of the old wound, with the strong membranous threads which attach it to the bone beneath; and then, secondly, to give such permanent support to the eyelid as will enable it to resist the contractile power of the granulations during the cicatrization of the wound below.

"The first indication is to be fulfilled by a single or double incision, from a little below the external to the internal angle of the eye, following the curve of the eyelid and of the orbit, as far and as deep as may be found sufficient for the removal of the cicatrix and of the cellular attachments to the bone beneath, which will be found exceedingly strong and firm, and sometimes requiring a dissection completely upon, or even within the edge of the orbit These adhesions are to be taken away, for, if only divided, they will reunite with greater firmness. The lid may then be raised to its proper situation, and every thread of cellular membrane, which seems to draw it downwards in the slightest degree, must be divided.

"The second indication was formerly attempted to be accomplished by supporting the eyelid by sticking plaisters placed across it, which answer very well until the cicatrization begins; but then they cease to offer a sufficient counteracting power, from the yielding nature of the parts to which they are attached; and the eyelid and the sticking plaisters are drawn down together. What is wanting, then, is evidently an opposing force, or counteraction, in a different direction, which shall be competent to prevent the contraction of the granulations in the wound, and the drawing in of the surrounding skin, which I have already shown in the instance of burns in the neck may be done, by great care and attention, in keeping the head backwards.

"This opposing force was sought for by Bordenave, in the removal of a fold of the conjunctiva from the inside of the lid; but has seldom or never, in cases of severity, been found sufficient; and when both operations were repeated and assisted in every way, as in the case of Professor Dzondi, they were only effectual with a submission to pain and inconvenience, which can scarcely be expected from any person.

"The removal of an angular portion of the lid, as proposed by Sir William Adams, and more especially in addition to the removal of the horizontal fold of conjunctiva, gives the opposing power required, in a degree quite sufficient to restrain the cicatrizing process from drawing the surrounding skin downwards, and a cure is therefore speedily and effectually accomplished. The removal of a V-like piece from the eyelid, and the approximation of the edges of the wound by sutures, shorten the part considerably and bind it over the eyeball: they alter the vicious curvature of the cartilage, and bring it nearly to a straight line, the object formerly desired to be obtained by the application of plaisters. This straightening of the eyelid which is effected in cartilaginous, ligamentous, and comparatively unyielding parts of the eyelid, is also permanent for a period equal to the contractile power *inherent* in the skin and granulations for a *certain* time after the cicatrix is completed; and therefore, the powers being equal, or rather against the cicatrix, the healing of that part is rapidly effected by the granulations filling up the wound, and the skinning process covering it in, by the formation of a new membrane, and not at the expense of the surrounding old skin.

"That the effect is produced by shortening the eyelid beyond, and not by merely restoring it to its natural state, is proved by the fact, that the eyelid, in its natural state, never offers a resistance equal to withstand the cicatrizing process below; of this, the disease itself is proof, as it could not otherwise have occurred; and therefore restoring it to its natural state only, will have no influence in promoting a cure, further than is gained by the cicatrization in a different direction. That this is the true principle on which the operation can be supported, will, I believe, be conceded by all those who are capable of estimating the value of opposing powers; and in performing the operation, it must be borne in mind by the surgeon, who will therefore cut out just as much as will bring the eyelid into this state, and yet not cause so great a separation of the edges of the wound, that the sutures cannot bring it together, without such a degree of extension as will prevent the union of the parts by the adhesive process. This portion to be removed may be taken out as far inwards as the middle of the tarsal cartilage when necessary, of which Case the 6th is an example; but it is in general more advantageously done nearer to the external angle, and the quantity to be removed will not, I believe, or as far as I know, ever exceed one quarter, and seldom more than an eighth, or from that to three sixteenths of an inch. The depth of the incision must be in proportion to the width of the part removed at the ciliary margin of the lid, in every case however extending beyond the cartilage. The operation for the third species consists, first, in the removal of the adhesions which the cicatrix has formed to the cheek-bone, with the whole or such part of the cicatrix as can be with propriety taken away; secondly, in the cutting out of an angular portion of the eyelid, near the external canthus; thirdly, in the removal of the diseased fold of the conjunctiva (if there be any), by a horizontal stroke of the scissors, assisted by the forceps; fourthly, in the application of two sutures, to bring the divided edges of the eyelid together; one close to the margin of the lid, the other near the point of the angle below; these are to be drawn just so tight as to bring the parts in close apposition, when they are to be cut off close to the knots, and supported by slips of sticking plaister; fifthly, in the dressing of the wound made by the removal of the adhesions to the bottom with very fine lint, and applying a bandage over the whole." 77.

The subsequent dressings require attention. The object intended by the sutures will almost always be effected by the third day, when the edges of the angular wound will be seen to adhere, and the sutures are consequently of no more service. They are therefore to be cut, and the threads removed. Strips of adhesive plaister should be applied in their place, with a compress and suitable support. Cases follow in elucidation of the text, for which we must refer to the work.

This eversion of the upper eyelid is a much less common disease than that of the under. It usually occurs in consequence of the contraction which takes place on the formation of a cicatrix, or from an abscess within, or a disease of the edge of the orbit. The effects of this disease are analagous to those already described, and lead, in the same manner, to the loss of sight. The attempts at cure do not always succeed.

The operation required for the cure of eversion of the upper eyelid, caused by cicatrization of an ulcer, is so analogous to that of the lower lid, that nothing need be said in detail on that subject.

Here we must close the first article of our analysis, as our limits do not permit us to do justice to such an important volume, without reserving a portion of its contents for another number of our journal. We have laid before the reader enough already to convince him that, in Mr. Guthrie's work, a great mass of valuable practical information will be found. The talents, the zeal, and the ample experience of the distinguished author insure any thing from his pen a general and extensive perusal.

